# A Review on Automatic Personal Identification Using Panoramic Radiographs and Computed Tomography

**DOI:** 10.1055/a-2808-8851

**Published:** 2026-03-04

**Authors:** Andreas Heinrich

**Affiliations:** 1 Institute for Diagnostic and Interventional Radiology 39065 Jena University HospitalJenaThuringiaGermany

**Keywords:** Artificial Intelligence, Computer Vision, forensics, Panoramic Radiography, Human Identification

## Abstract

**Background:**

Identifying completely unknown individuals is a major challenge in forensic and emergency medicine. Radiology offers a promising solution by using unique anatomical features on medical images to identify both living and deceased persons. Although emergency or postmortem images could be matched against large clinical databases, such applications remain largely experimental. This review examines current methods in automatic radiology-based personal identification, evaluates their performance, and highlights potential applications in forensic and clinical settings.

**Method:**

A narrative review of studies published from 2018 onwards was conducted using PubMed and Google Scholar. Included studies applied automated or semi-automated personal identification to panoramic radiographs (PR) or computed tomography (CT) using reference datasets. A narrative approach was used to synthesize results descriptively due to heterogeneity in study design, dataset size, and methodology.

**Results:**

Of the 32 included studies, 15 focused on PR-to-PR, 8 on head CT-to-CT, 7 on body CT-to-CT, and 2 on CT-to-PR identification. The most commonly applied approach was descriptor-based computer vision (CV), used in 9 studies. Deep learning was applied in 8 studies for feature extraction, and in 2 studies each for classification and bone segmentation.

**Conclusion:**

Several methods perform well in controlled settings. Descriptor-based CV provides the most flexibility and strongest evidence, especially for large-database comparisons and postmortem applications. Deep learning approaches, including feature extraction, classification, and automatic bone segmentation, also show promise for cross-individual matching but require further validation. Automatic radiology-based personal identification holds significant potential for forensic and clinical use, yet the development of standardized large-scale reference databases and robust automated pipelines remains a key challenge.

**Key Points:**

- Radiological images enable automated personal identification of unknown individuals.
- Descriptor-based computer vision is flexible and robust for large database matching.
- Deep learning shows promise for cross-individual matching, but requires further validation.
- Postmortem applications are feasible, yet under-investigated.
- Ethical frameworks are necessary for handling sensitive imaging data.

**Citation Format:**

- Heinrich A. A Review on Automatic Personal Identification Using Panoramic Radiographs and Computed Tomography. Rofo 2026; DOI 10.1055/a-2808-8851

## Zusammenfassung

**Hintergrund:**

Die Identifikation vollständig unbekannter Personen stellt nach wie vor eine große Herausforderung in der Rechts- und Notfallmedizin dar. Die Radiologie bietet einen vielversprechenden Ansatz, indem sie einzigartige anatomische Merkmale in medizinischen Bildern nutzt, um sowohl lebende als auch verstorbene Personen zu identifizieren. Obwohl Notfall- oder postmortale Bilder grundsätzlich mit großen klinischen Datenbanken abgeglichen werden könnten, sind solche Anwendungen weitgehend noch experimentell. Dieses Review untersucht aktuelle Methoden der automatischen Radiologie-basierten Personenidentifikation, bewertet ihre Leistungsfähigkeit und beleuchtet mögliche forensische und klinische Anwendungen.

**Methode:**

Es wurde eine narrative Übersichtsarbeit zu Studien ab dem Jahr 2018 unter Verwendung von PubMed und Google Scholar durchgeführt. Eingeschlossene Studien wendeten automatisierte oder halbautomatisierte Personenidentifikationsverfahren auf Panoramaröntgenaufnahmen (PR) oder Computertomografie (CT) anhand von Referenzdatensätzen an. Aufgrund der Heterogenität in Studiendesign, Datensatzgröße und Methodik wurden die Ergebnisse narrativ und deskriptiv zusammengefasst.

**Ergebnisse:**

Von den 32 eingeschlossenen Studien beschäftigten sich 15 mit PR-zu-PR, 8 mit Kopf-CT-zu-CT, 7 mit Körper-CT-zu-CT und 2 mit CT-zu-PR-Identifikation. Der am häufigsten angewandte Ansatz war die Deskriptor-basierte Computer Vision (CV) und wurde in 9 Studien eingesetzt. Deep Learning kam in 8 Studien zur Merkmalsextraktion sowie in jeweils 2 Studien zur Klassifikation und Knochen-Segmentierung zum Einsatz.

**Schlussfolgerung:**

Mehrere Methoden erzielen gute Ergebnisse unter kontrollierten Bedingungen. Deskriptor-basierte CV bietet die größte Flexibilität und die stärksten Evidenzen, insbesondere für Vergleiche mit großen Datenbanken und für postmortale Anwendungen. Deep Learning-Ansätze, einschließlich Merkmalsextraktion, Klassifikation und automatischer Knochensegmentierung, zeigen ebenfalls Potenzial für den Abgleich zwischen Individuen, benötigen jedoch weitere Validierung. Die automatische Radiologie-basierte Personenidentifikation bietet erhebliches Potenzial für die forensische und klinische Praxis, doch bleibt die Entwicklung standardisierter, groß angelegter Referenzdatenbanken und robuster automatisierter Pipelines eine zentrale Herausforderung.

**Kernaussagen:**

- Radiologische Bilder ermöglichen automatisierte Personenidentifikation unbekannter Identitäten.
- Deskriptor-basierte Computer Vision ist flexibel und robust für große Datenbankabgleiche.
- Deep Learning zeigt Potenzial für Vergleiche zwischen Individuen, benötigt weitere Validierung.
- Postmortale Anwendungen sind möglich, aber noch wenig erforscht.
- Ethische Rahmenbedingungen sind für den Umgang mit sensiblen Bilddaten notwendig.

## Introduction

Identifying unknown individuals after accidents, natural disasters, terrorist attacks, armed conflicts, migration, or in cases of homelessness is a major challenge in forensic and emergency medicine 1 2 3 4 . Traditional methods such as fingerprints, deoxyribonucleic acid (DNA) analysis, and odontological identification are reliable but depend on the availability of reference material 5 . Without any clues to the individual’s identity, whether from personal belongings, witnesses, or distinguishing physical traits, no suitable reference material is available for comparison, making rapid identification impossible. Radiological imaging is an underused resource for personal identification, as anatomical structures in panoramic radiographs (PRs) and computed tomography (CT) scans exhibit substantial interindividual variability 6 7 . Increasing digitization enables automatic comparison of emergency or postmortem images with large clinical image databases from hospitals, clinics, and dental practices. To exploit this potential, computer vision (CV) techniques, including traditional mathematically interpretable methods as well as newer artificial intelligence (AI) based approaches, particularly convolutional neural networks (CNNs), can detect, analyze, and interpret visual information, enabling automated personal identification. This narrative review summarizes current developments in radiological personal identification using PR and CT. It focuses on automated and semi-automated methods for matching with reference data, compares approaches across different anatomical regions and algorithms, and outlines current challenges as well as directions for future research.

## Review methodology

This narrative review synthesizes recent evidence on methods for identifying unknown individuals by comparing their radiological data with large antemortem reference datasets using PRs and CT. The literature search was performed in PubMed and Google Scholar and was complemented by screening the reference lists of relevant studies. The focus was on studies reporting practical implementation of automated or semi-automated radiological identification methods. Studies were included if they used PR or CT, were published from 2018 onwards, applied an identification method with a reference dataset, and were peer-reviewed original research with accessible full text. The exclusion criteria encompassed case reports, conference abstracts, and studies with major methodological limitations (e.g., deep learning approaches without independent test datasets). Relevant studies were screened and full texts reviewed. The data that was collected included publication year, anatomical region, methods, dataset characteristics, and performance metrics. Rank accuracy reflects retrieval performance: rank-1 indicates the top match is correct, while rank-10 means the correct individual appears within the top 10 matches. Due to heterogeneity in study designs, datasets, and outcome reporting, a formal meta-analysis was not feasible. Therefore, the results were synthesized descriptively.

## Findings

Among the 32 included studies, traditional CV (12 studies), AI-based CV approaches (16 studies), and 4 rule-based methods were predominantly applied ( Fig. 1 ). Table 1 summarizes the key methods and the number of studies implementing each method. The following sections present the main findings across different imaging modalities and anatomical regions.

**Fig. 1:**
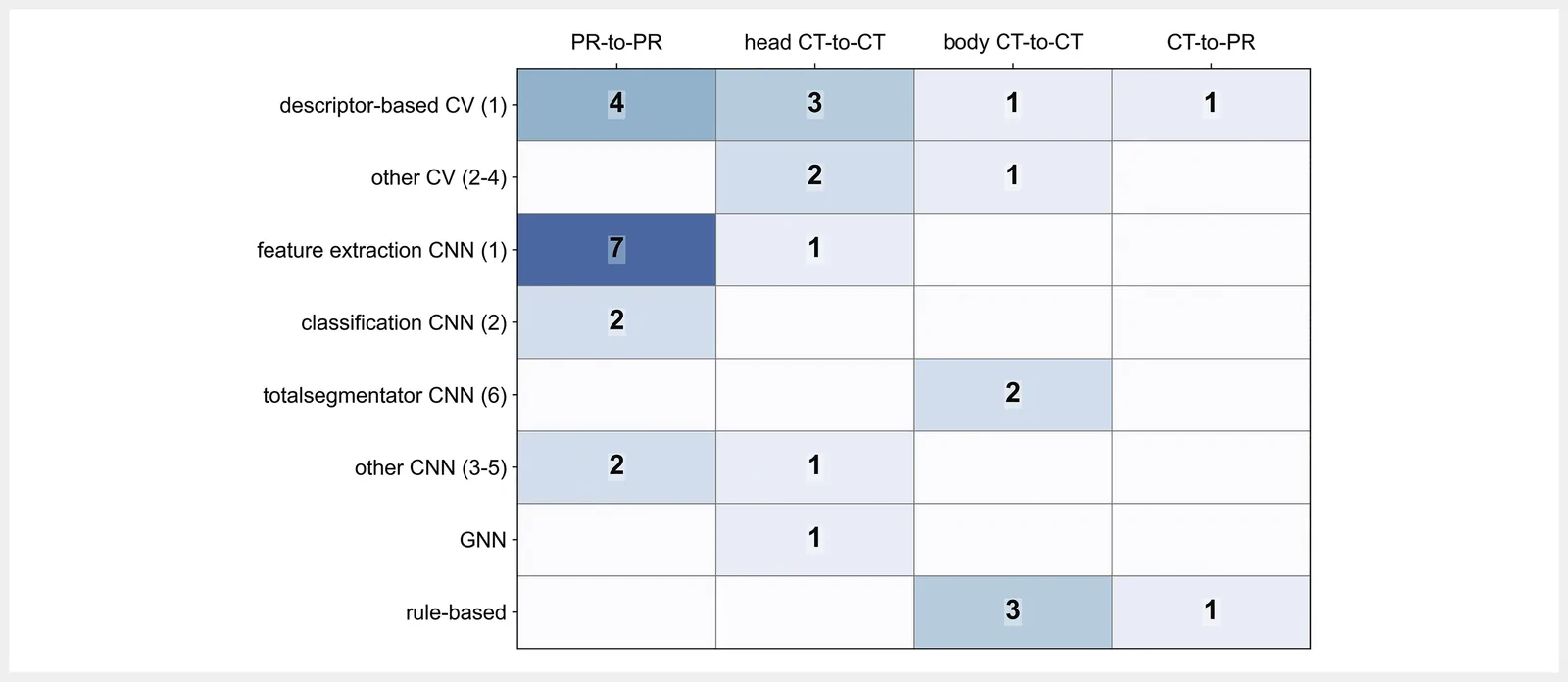
Number of studies per method and imaging modality for automated personal identification. Method details are provided in Table 1 .

**Table:** **Table 1** Overview of traditional computer vision (CV), AI-based CV using convolutional neural network (CNN), and graph neural network (GNN) methods for automated personal identification, with n indicating the number of studies using each method.

Type		n	Method	Explanation
CV	1	9	Descriptor-based	Automatically detects distinctive points (key points) in an image. Each key point has a descriptor that describes the local texture, like a fingerprint. Descriptors from different images are compared to find matches. Example algorithms include Speeded-Up Robust Features (SURF) and Accelerated-KAZE (AKAZE).
	2	1	Beam Angle Statistics (BAS)	Describes the shape of a structure’s boundary by measuring angles along its outline. These measurements are combined into a feature vector that can be compared across images.
	3	1	Iterative Closest Point (ICP)	Aligns two 3D shapes by matching closest points and adjusting their positions. The resulting alignment error tells how similar the shapes are.
	4	1	Template matching	Compares small regions of one image to the same regions on another image to find matches. Works best if scale, rotation, and lighting are similar.
CNN	1	8	Feature extraction	Uses deep learning to automatically learn complex patterns, shapes, and textures in images. The network must be trained with many examples to identify the most distinctive visual traits. After training, new images are converted into compact numerical feature vectors summarizing the whole image, which are compared across individuals for identification.
	2	2	Classification	Trains a neural network on labeled images to directly assign a new image to a known individual. This works best when many images per individual are available and can only identify people included in the training set.
	3	1	Object detection	Uses a CNN (e.g., EfficientDet‑D3) to detect and label specific structures or landmarks, such as teeth, implants, or root canal treatments. Detected objects can be compared to reference images (e.g., presence, type, position) for identification.
	4	1	Siamese network	A neural network that processes two images simultaneously to measure their similarity. During training, it learns which visual patterns indicate a match. In the case of inference, it can compare new images to references and determine whether they belong to the same individual, even if the person was not in the training set.
	5	1	Stacked Convolutional Autoencoder (SCAE)	An unsupervised method that compresses images into compact feature representations by encoding and then reconstructing them. The resulting low-dimensional features capture the most relevant visual patterns and can be compared across images without labeled data.
	6	2	TotalSegmentator	Automatically segments over 100 anatomical structures on 3D CT images (bones, organs, muscles, vessels, etc.). The segmented models can be compared to reference scans for identification.
GNN	1	1	Geometric Self-Attention Network (GSA-Net)	Compares 3D point clouds using a graph-based neural network that captures both local and global geometric patterns. Generates robust feature representations that can be compared across individuals, even if the data is partially missing or differently oriented.

### PR-to-PR identification

This section summarizes 15 studies on automated PR identification ( Table 2 ).

**Table:** **Table 2** Overview of studies on automated personal identification from PRs. Query (Q) indicates the number of test individuals or samples, and Reference (REF) indicates the number of reference individuals or samples in the dataset. When “other” methods were tested, only the approach with the best results is listed. The asterisk (*) indicates that the study used 5-fold cross-validation, and the data shown corresponds to a single fold. Abbreviations are explained in Table 1 .

Studies	Method		Individuals		Samples		Rank [%]	
	Type	Details	Q	REF	Q	REF	1	10
Heinrich et al. 2018 8	CV (1)	SURF on preprocessed edge-enhanced images	40	24545	40	43467	85	90
Heinrich et al. 2020 9	CV (1)	SURF on preprocessed edge-enhanced images, 3 postmortem queries	43	33206	43	61545	100	100
Fan et al. 2020 10	CNN (1)	DENT‑net for feature extraction; trained on 15369 PRs (6300 individuals)	173	173	326	173	85	~100
Lai et al. 2020 11	CNN (1)	LCANet for feature extraction; trained on 22172 PRs (9490 individuals)	503	503	665	503	87	95 (rank 5)
Wu et al. 2021 12	CNN (1)	AMNet for feature extraction; trained on 22172 PRs (9490 individuals)	503	503	665	503	89	96 (rank 5)
Ortiz et al. 2021 13	CNN (1)	Inception v3 for feature extraction; no additional training	100	100	100	100	85	–
Kim et al. 2021 14	CNN (2)	Modified VGG16 for classification; trained on 2014 PRs (746 individuals)	746	746	746	746	83	92 (rank 5)
ATAŞ et al. 2022 15	CNN (1)	PDR-net for feature extraction; trained on 600 PRs (300 individuals)	72	72	72	143	85	98
Chen et al. 16	CNN (1)	Fine-grained bilateral-branch network for feature extraction; trained on 21575 PRs (9113 individuals)	200*	200*	~228*	200*	89	96
Enomoto et al. 2022 17	CNN (2)	VGG16 (+others) for classification; trained on 3303 PRs (1663 individuals)	1663	1663	1663	1663	66	86
Lin et al. 2023 18	CNN (1)	GAN framework with a CNN-based classifier for feature extraction; trained on 22172 PRs (9490 individuals)	503	503	665	503	93	–
Heinrich 2024 19	CV (1)	AKAZE on preprocessed edge-enhanced images	70	56008	70	105251	100	100
Choi et al. 2024 20	CNN (3)	EfficientDet-D3 to detect teeth, numbers, and restorations for similarity matching; trained on 1638 PRs	1029	1029	1029	1029	60 (rank 51)	
Bozkurt et al. 2025 21	CV (1)	SURF (+others) on segmented mandibular teeth	250 (split not specified)				91	100
Pereira et al. 2025 22	CNN (4)	VGG16 for classification as Siamese network, pairwise PR comparison (match/no match); trained on 923 PRs	43	–	43	164	67	–

Four studies 8 9 19 21 used descriptor-based CV on preprocessed images ( Fig. 2 ). Heinrich et al. 8 9 19 evaluated 40–70 query individuals against 43467–105251 reference PRs, achieving rank-1 accuracies of 85–100%. Of the 15 studies reviewed, only one 9 investigated postmortem cases, successfully matching three PRs to a large antemortem database. Another study 21 also applied descriptor-based CV, using tooth segmentation prior to matching. Despite limited details on dataset splitting, these results demonstrate the robustness of descriptor-based CV for PR identification.

**Fig. 2:**
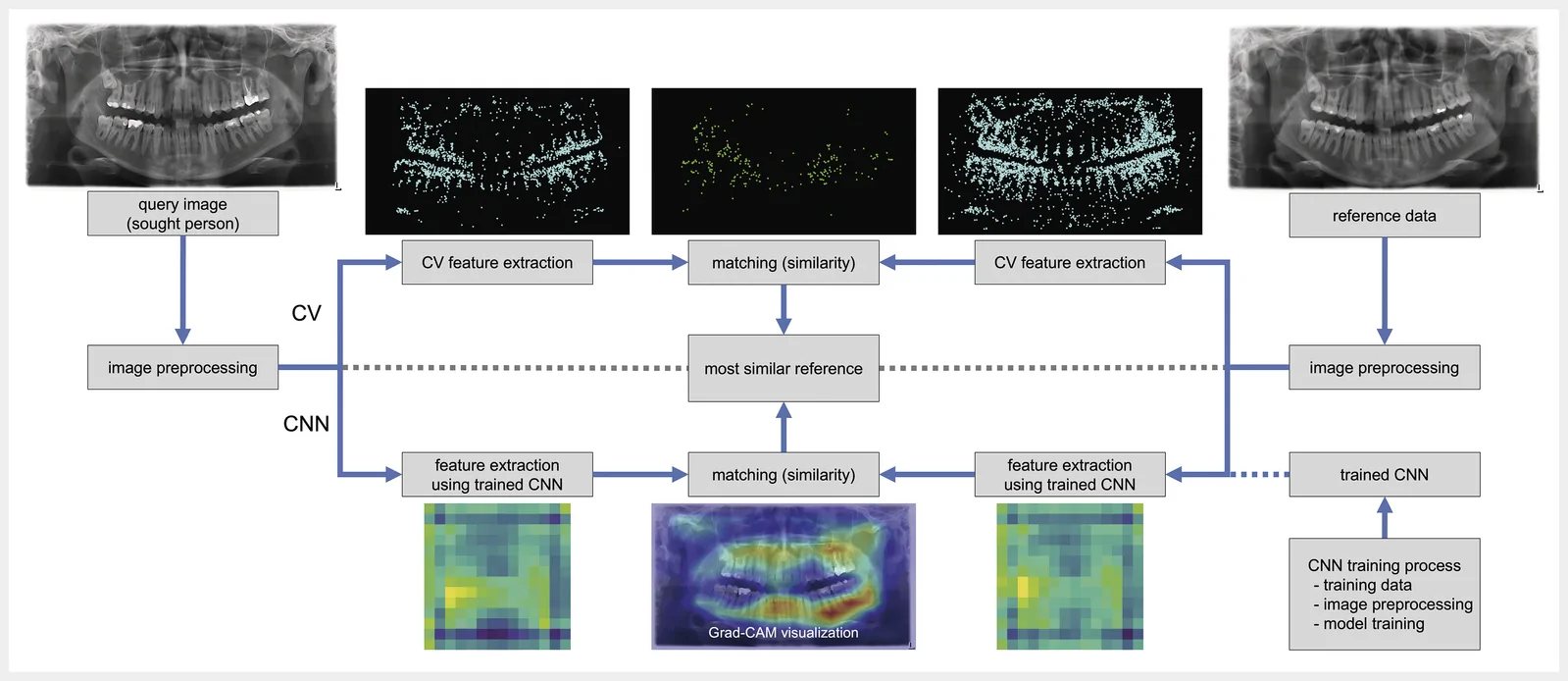
Comparison of descriptor-based CV (top) and CNN-based feature extraction (bottom) for automated personal identification. Descriptor-based CV compares individual key points, requiring only local image fragments to match. CNN extracts global features from the entire image and requires prior training for the specific modality. Grad-CAM can highlight which image regions contribute most to feature activation, but provides only a visual guide without quantitative or causal interpretation.

The remaining 11 studies applied CNN-based approaches, following four distinct methodological strategies. Seven studies 10 11 12 13 15 16 18 applied CNNs for feature extraction to learn generalizable representations that could subsequently be compared across PRs. Ortiz et al. 13 used a pre-trained Inception v3 network without additional PR-specific training, while the others 10 11 12 15 16 18 trained networks on 600 to 22172 PRs. Despite differences in training strategies and dataset sizes, rank-1 accuracy remained around 85%, suggesting CNN feature extraction is robust across scales. However, the corresponding query and reference datasets were relatively small, with 72 to 665 PRs for query PRs and 100 to 503 PRs for reference PRs. Chen et al. 16 extracted features from both whole PRs and individual tooth masks, fusing them for identification, and evaluated their model in a 5-fold cross-validation setup, with each fold using approximately 228 query PRs against 200 reference PRs, achieving a rank-1 accuracy of 89%. Lin et al. 18 extended this approach by using a generative adversarial network (GAN) to augment feature learning, while the final classification still relied on a CNN-based classifier for feature extraction, achieving a slightly higher rank-1 accuracy of 93% with 665 query and 503 reference PRs. Two studies 14 17 trained CNNs for direct classification of individual identities, assigning each query image to a known individual. Enomoto et al. 17 trained on a larger number of individuals with fewer images per person and achieved moderate rank-1 accuracy (66%), whereas Kim et al. 14 trained on fewer individuals with more images per person, reaching higher rank-1 accuracy (83%). These results suggest that both the number of individuals and the number of training PRs per individual can influence performance. Choi et al. 20 employed a CNN-based object detection approach using EfficientDet‑D3 to identify teeth, numbers, restorations, and other dental structures, generating a similarity score for each potential match. Performance was reported only as an approximate frequency of the target individual appearing among the top 5% of candidates, which is why Table 2 lists a rank of 51 for 1,029 reference images. Finally, Pereira et al. 22 applied a Siamese network based on VGG16, comparing pairs of PRs to classify them as match or non-match. The model was trained on 923 PRs and tested on 43 query PRs against 164 reference PRs, achieving a rank-1 accuracy of 67%.

### CT-to-CT identification

This section summarizes the main approaches and performance metrics of 15 studies investigating automated identification using CT images.

#### Head

Eight studies specifically addressed head CT for automated identification ( Table 3 ), employing a variety of methods.

**Table:** **Table 3** Overview of studies on automated personal identification from head CT. Query (Q) indicates the number of test individuals or samples, and Reference (REF) indicates the number of reference individuals or samples in the dataset. When “other” methods were tested, only the approach with the best results is listed. Abbreviations are explained in Table 1 .

Studies	Method		Individuals		Samples		Rank [%]	
	Type	Details	Q	REF	Q	REF	1	10
De Souza et al. 2018 23	CV (2)	BAS on automatic frontal sinus segmentation	31	31	31	31	77	98
Souadih et al. 2019 24	CNN (5)	SCAE for feature extraction on automatic 3D sphenoid sinus segmentation; trained on 20 images	13	70	13	70	100	100
Wen et al. 2022 25	CNN (1)	MVSS-Net for feature extraction on semiautomatic 3D sphenoid sinus segmentation; trained on 1203 datasets (600 individuals)	132	132	141	132	94	100
Dong et al. 2022 26	CV (3)	ICP for 3D point cloud matching of semiautomatic 3D sphenoid sinus models	743	732	743	732	96	100
Li et al. 2024 27	GNN (1)	GSA‑Net for 3D point cloud matching of semiautomatic 3D sphenoid sinus models; trained on 1035 models (512 individuals)	220	220	220	220	100	100
Heinrich 2024 28	CV (1)	AKAZE on preprocessed edge-enhanced images of six regions (best maxillary sinuses)	69	719	69	815	97	100
Heinrich et al. 2025 29	CV (1)	AKAZE on preprocessed edge-enhanced images of maxillary sinuses; 10 postmortem queries	10	738	232	60255	50	100
Torimitsu et al. 2025 30	CV (1)	AKAZE (+other) on manually preprocessed frontal sinuses; 180 postmortem queries	180	180	180	180	49	77

Three distinct traditional CV approaches have been published for head CT identification 23 26 28 29 30 . De Souza et al. 23 applied beam angle statistics (BAS) to frontal sinus segmentations, achieving a rank-1 accuracy of 77% on a very small dataset of 31 query and 31 reference CT scans. Dong et al. 26 used iterative closest point (ICP) matching on semiautomatically segmented 3D sphenoid sinus models. Despite the manual preprocessing required to generate the point clouds, the method achieved high accuracy, with a rank-1 identification rate of 96% across 743 query and 732 reference CT scans. Heinrich 28 demonstrated that descriptor-based CV could achieve 97% rank-1 accuracy with only a single CT slice of the maxillary sinus, comparing 69 query slices against 815 reference images. In a subsequent study 29 , the approach was extended to postmortem CT scans, showing that a rank-1 identification rate of 50% can be achieved when 10 postmortem cases are matched against a clinical database comprising 738 individuals with 60,255 reference images. Torimitsu et al. 30 also demonstrated that descriptor-based CV can be applied to postmortem CT images, achieving a rank-1 accuracy of 49% for 180 postmortem frontal sinus images. However, this approach required manual alignment and optimization of the postmortem images to match the antemortem scans.

The AI-based CV approaches 24 25 27 focused exclusively on semiautomatically and automatically segmented 3D sphenoid sinus data. Souadih et al. 24 used a stacked convolutional autoencoder (SCAE) for feature extraction from automatically segmented 3D sphenoid sinus volumes, trained on 20 images, achieving 100% rank-1 accuracy on 13 query and 70 reference CT scans. Wen et al. 25 employed MVSS-Net for feature extraction on semiautomatically segmented 3D sphenoid sinus reconstructions, trained on 1,203 instances from 600 individuals, reaching 94% rank-1 accuracy with 141 query and 132 reference scans. Li et al. 27 applied a graph neural network (GNN) approach using a geometric self-attention network (GSA-Net) for 3D point cloud matching of semi-automatically segmented sphenoid sinus models, achieving 100% rank-1 accuracy on 220 query and 220 reference scans.

#### Body

The body CT-based approaches comprised rule-based methods, traditional CV techniques, and two CNN-assisted segmentation approaches used solely for 3D segmentation ( Table 4 ).

**Table:** **Table 4** Overview of studies on automated personal identification from body CT. Query (Q) indicates the number of test individuals or samples, and Reference (REF) indicates the number of reference individuals or samples in the dataset. MIP stands for maximum intensity projection. Abbreviations are explained in Table 1 .

Studies	Method		Individuals		Samples		Rank [%]	
	Type	Details	Q	REF	Q	REF	1	10
Weiss et al. 2018 31	–	Manual measurement of 12 sternal bone features by two readers; automated comparison of features; 44 postmortem queries	44	44	44	94	65	77
Decker et al. 2018 32	–	Semiautomated 3D part-to-part lumbar vertebra comparison; manual landmarks, automated alignment, percent-match similarity	30	30	30	30	100	100
Ueda et al. 2019 33	CV (4)	Template-matched local maxima on corrected scout images	619	619	619	619	100	100
Sato et al. 2022 34	–	Manual measurement of shortest diameter of thoracic vertebrae (T1-T12); automated comparison; 82 postmortem queries	82	702	82	702	99	100
Neuhaus et al. 2025 35	CNN (6)	TotalSegmentator for 3D segmentation of sternal bone and T5 vertebra; comparison of two aligned 3D models using the Dice coefficient; 40 postmortem queries	40	90	40	90	98	–
Heinrich et al. 2025 36	CV (1)	AKAZE on preprocessed thoracic-MIP images	300	8177	300	12465	99	100
Ichikawa et al. 2025 37	CNN (6)	TotalSegmentator for segmentation of thoracic vertebral bodies (T1-T12); distance-based 3D shape comparison	61	1041	61	1041	98	100

Three studies 31 32 34 did not employ CV methods but demonstrated the potential of bone-based identification. Weiss et al. 31 performed manual measurements of 12 sternal bone features by two readers, followed by automated comparison, achieving 65% rank-1 accuracy across 44 postmortem query and 94 reference scans. Decker et al. 32 conducted semi-automated 3D part-to-part comparisons of lumbar vertebrae using manually placed landmarks and automated alignment, achieving 100% rank-1 accuracy on 30 query and 30 reference scans. Sato et al. 34 measured the shortest diameters of thoracic vertebrae manually, followed by automated comparison, achieving 99% rank-1 accuracy for 82 query and 702 reference scans.

Two different traditional CV methods were applied. Heinrich et al. 36 applied descriptor-based CV to thoracic maximum intensity projection (MIP) images, achieving 99% rank-1 accuracy across 300 queries and 12465 reference scans. Ueda et al. 33 applied template matching to local maxima extracted from geometrically corrected scout CT images. This approach compared small regions across images to identify individuals, achieving 100% rank-1 accuracy on 619 query and 619 reference scans.

Two CNN-assisted methods were reported, both using TotalSegmentator 38 solely for fully automated 3D segmentation, without employing AI for identification or comparison. Neuhaus et al. 35 performed pairwise Dice coefficient comparisons of aligned sternal and T5 vertebra segmentations, achieving 98% rank-1 accuracy across 40 postmortem queries and 90 reference scans. Ichikawa et al. 37 performed distance-based 3D shape comparisons of thoracic vertebral bodies, achieving a rank-1 accuracy of 98% across 61 postmortem query and 1041 reference scans.

### CT-to-PR identification

Two studies investigated cross-modal identification between PR-like images reconstructed from CT scans and PRs ( Table 5 ). Fujimoto et al. 39 focused on the alveolar bone and performed all landmarking manually with semi-automatic preprocessing. Deep learning was only mentioned as a potential future approach. Comparability is limited, as rank-based accuracy metrics were not reported. In contrast, Heinrich’s group demonstrated fully automated cross-modal identification using descriptor-based CV 40 , achieving 72% rank-1 accuracy across 50 query cases and 82036 reference PRs.

**Table:** **Table 5** Overview of studies on automated personal identification using PR-like images derived from CT data. PR-like images are created from curved multiplanar reconstructions (MPRs) to resemble PR and are compared with actual PRs for identification. Query (Q) indicates the number of test individuals or samples, and Reference (REF) indicates the number of reference individuals or samples in the dataset. The asterisk (*) means that the study divided the data into three groups. Abbreviations are explained in Table 1 .

Studies	Method		Individuals		Samples		Rank [%]	
	Type	Details	Q	REF	Q	REF	1	10
Fujimoto et al. 2022 39	–	Manual landmark placement on alveolar bone with pairwise Procrustes comparison of manually generated PR-like images and PRs; postmortem queries	160*	–	160*	357*	–	–
Woitke et al. 2025 40	CV (1)	AKAZE on preprocessed edge-enhanced images of automatically generated PR-like images and PRs	50	43379	50	82036	72	82

## Discussion

Automatic radiology-based methods for personal identification hold strong promise for both forensic and clinical applications. However, current studies show substantial methodological heterogeneity. The included studies vary widely with regard to anatomical region, preprocessing procedures, dataset size, and composition (mostly adults, children are present in 9 ), and identification strategies. This variability limits direct comparability and complicates broader conclusions about the generalizability and real-world applicability. Table 6 compares the most common methods by function, error susceptibility, and strengths.

**Table:** **Table 6** The table presents the functioning, possible causes of false positives and false negatives, as well as the strengths and limitations of methods applied in at least two studies. Table 1 for details regarding the methods.

Method	Principle	Compared features	Training needed	False positives	False negatives	Strengths	Limitations
CV (1)	Local key point descriptor matching; identity indicated by number of matching points	Local patterns around key points	No	Similar local structures (e.g., labels, highly similar anatomical edges, large standardized implants)	Major global anatomical changes (e.g., growth, new large implants); variation in image acquisition (e.g., closed mouth obscuring key edges, angle)	Universally applicable; robust to changes in position, size, rotation, and intensity; suitable for all ages, postmortem cases, and selected cross-modal scenarios; masking irrelevant regions improves reliability	Limited global context; performance depends on number and quality of key points
CNN (1)	Learned global image representation via CNN; identity indicated by similarity of feature vectors	Whole-image feature vectors	Yes	Similar global structures (e.g., global anatomical resemblance, large standardized implants)	Anatomical changes (e.g., growth, tooth loss, implants, surgery); variations in image acquisition (e.g., pose, zoom, angle, intensity)	Captures complex global patterns; flexible feature learning	Sensitive to appearance changes; requires large, representative training data; limited interpretability; affected by domain shifts
CNN (2)	Direct classification into predefined identity classes	Identity labels (known individuals only)	Yes (per identity)	Confusion between individuals with similar anatomy or dental patterns	Anatomical changes not represented during training (e.g., growth, tooth loss, implants, surgery); variations in image acquisition (e.g., pose, zoom, angle, intensity)	Captures complex global patterns; simple inference once trained	Strongly dependent on training data composition; cannot generalize to unseen individuals
CNN (6)	Automatic segmentation followed by 3D shape comparison	Segmented anatomical models	Yes	Segmentation artifacts; partial or incorrect delineation	Poor image quality; incomplete scans; trauma or postoperative changes	Anatomically interpretable; structure-based comparison	Errors propagate directly from segmentation; sensitive to anatomical alterations

In PR-to-PR identification, three methodological trends stand out as particularly promising in terms of accuracy and robustness. The first comprises descriptor-based CV 8 9 19 , which consistently demonstrates reliable identification performance even when matched against very large antemortem databases, in one case exceeding 100000 reference PRs, suggesting potential scalability beyond currently tested database sizes. These approaches have also been successfully extended to postmortem cases. Because they rely on comparing local key points rather than on a trained model, they can make effective use of small anatomical regions; a single distinctive tooth or restoration may be sufficient for a correct match. Descriptor-based CV is largely invariant to scaling, rotation, variations in signal intensity, image noise, and, to some extent, perspective distortion 9 . The second methodological trend is CNN-based feature extraction 10 11 12 13 15 16 18 . These methods encode the entire PR into numerical feature representations that capture complex spatial patterns across the dentition. This allows highly accurate matching, even when subtle structures provide discriminative information. However, the resulting feature vectors may be sensitive to temporal changes such as tooth loss, restorations, or implants, which can reduce cross-time comparability. To address this, Chen et al. 16 additionally incorporated features from segmented tooth masks, fusing them with whole-image representations to improve robustness against individual variations and temporal changes. The third trend is CNN-based classification 14 17 . These models learn to assign each PR to a specific individual. Their performance generally improves with a larger number of training images per person, but the approach is inherently constrained by the number of individuals the network can handle as classes. Consequently, scalability to large database sizes remains challenging, and applicability to open-set identification scenarios is limited. Other approaches, such as Siamese networks or object detection for teeth and restorations, showed conceptual promise but currently deliver less consistent performance.

In head CT-to-CT identification, a wide methodological variety has been applied, with consistently high accuracy reported across studies. Descriptor-based CV 28 29 30 again demonstrates strong potential, including for postmortem cases, particularly for comparisons of individual head slices, such as the maxillary sinuses, against large reference datasets. Other traditional CV approaches, such as ICP matching of semiautomatically segmented 3D sphenoid sinus models 26 , also achieve high accuracy and robustness, highlighting the versatility of traditional CV for structured anatomical comparisons. AI-driven approaches 24 25 27 , including CNN-based feature extraction and GNN methods for 3D point cloud matching, perform excellently with respect to the sphenoid sinus. These methods are highly effective when trained and applied to this specific anatomical region, capturing complex spatial patterns across 3D structures. However, their generalizability to other cranial regions or larger head CT datasets remains to be demonstrated. Nevertheless, they illustrate the potential of AI-based approaches to learn detailed geometric or volumetric features that are difficult to encode manually. Manual preprocessing currently limits the applicability of some methods 25 26 27 30 to very large datasets, but advances in automated segmentation are likely to mitigate these limitations in the future.

In body CT-to-CT identification, a variety of methods have been applied, consistently achieving very high accuracy. Descriptor-based CV 36 again demonstrated strong potential, achieving high identification performance on thoracic MIP images with a large reference database. Template-matching of local maxima on geometrically corrected scout images 33 also yielded promising results. However, this approach is less robust, as it relies on consistent image quality, scanner settings, and patient conditions, which may limit its applicability for large-scale database matching. Manual measurements of bones 31 32 34 show potential but require automation for large-scale application. The use of TotalSegmentator for the segmentation of selected anatomical structures, such as bones, followed by pairwise comparison between individuals represents a highly promising approach 35 37 . Its success, however, depends entirely on the quality of the automated segmentation, and further validation on large, heterogeneous reference datasets is needed to confirm its robustness and feasibility for practical identification scenarios.

In CT-to-PR identification, cross-modal comparisons remain relatively underexplored. Descriptor-based CV approaches 40 , applied to automatically generated PR-like images from CT data, have shown strong potential, achieving promising identification performance against large PR reference datasets. These findings indicate that descriptor-based CV can be successfully adapted for cross-modal scenarios, although further research is needed to evaluate their robustness and generalizability across larger and more diverse datasets.

Across all reviewed studies, descriptor-based CV provides the most consistent and robust evidence across PR 8 9 19 21 and CT 28 29 30 36 comparisons, including cross-modal identification 40 . Implementation is comparatively straightforward, as no model training is required, and the computations are transparent and interpretable. Several studies 8 9 36 identified a minimum threshold of matching key points above which an individual could be reliably identified, without the need for a full database comparison, making these methods particularly suitable for large-scale and postmortem applications. Efficiency and accuracy can be further improved by restricting comparisons to database entries with matching characteristics such as sex, age 19 41 , or estimated body weight 42 . Importantly, descriptors are abstract and cannot be used to reconstruct images. They also do not contain personal information 29 . This allows them to be compared across datasets without revealing identities. Re-identification is only possible if descriptors are linked to metadata, such as the data source or a pseudo-anonymized patient ID. This enables privacy-preserving automated identification while allowing controlled and ethical data linkage.

AI-based methods have mostly been applied to PR-to-PR comparisons, with head CT limited to the sphenoid sinus and body CT to segmentation via TotalSegmentator. Evaluated primarily on small, homogeneous datasets, their robustness across populations and scanner settings remains uncertain. Feature extraction encodes images or 3D structures into high-dimensional vectors, providing accurate matches but yielding representations that are difficult to interpret. Direct classification assigns images to known individuals, benefiting from larger training sets but limited to the classes included in training. CNN-assisted segmentation standardizes workflows but depends on segmentation quality. Despite these challenges, AI has strong potential to capture anatomical detail beyond traditional descriptors, though further work is needed to ensure generalizability, interpretability, and ethical application.

In conclusion, various methodological strategies exist, but small, homogeneous datasets limit confidence in their robustness across populations and scanner settings. Performance in controlled studies may overestimate real-world feasibility. Multicenter datasets and independent validations are still lacking. Despite these limitations, descriptor-based CV is closest to clinical and forensic translation. Privacy-preserving descriptors can be shared centrally while keeping original images local, offering a practical, ethical route for large-scale, cross-institutional identification. Radiology will play a key role in enabling such processes. However, it is essential to emphasize that automated techniques are not intended to replace forensic experts, but rather to streamline and support the search for suitable reference material.
